# A Novel 2-Hit Zebrafish Model to Study Early Pathogenesis of Non-Alcoholic Fatty Liver Disease

**DOI:** 10.3390/biomedicines10020479

**Published:** 2022-02-17

**Authors:** Abhishek Kulkarni, Sara Ibrahim, Isra Haider, Amina Basha, Emma Montgomery, Ebru Ermis, Raghavendra G. Mirmira, Ryan M. Anderson

**Affiliations:** 1 Department of Medicine, The University of Chicago, Chicago, IL 60637, USA; abhikulkarni@medicine.bsd.uchicago.edu (A.K.); amina.basha@uchospitals.edu (A.B.); ejmontgomery@uchicago.edu (E.M.); eermis@uchicago.edu (E.E.); 2 The Center for Diabetes and Metabolic Diseases, Indiana University School of Medicine, Indianapolis, IN 46202, USA; saibrahi@iupui.edu (S.I.); inhaider@iu.edu (I.H.)

**Keywords:** NAFLD, inflammation, zebrafish, obesity, PIC3, macrophages, models, screening, pioglitazone

## Abstract

Nonalcoholic fatty liver disease (NAFLD) is one of the most common liver diseases in adults. NAFLD progresses from benign liver fat accumulation to liver inflammation and cirrhosis, and ultimately leads to liver failure. Although several rodent models have been established for studying NAFLD, they have limitations that include cost, speed of disease development, key dissimilarities, and poor amenability to pharmacological screens. Here, we present a novel 2-hit zebrafish model to replicate aspects of NAFLD pathogenesis. We fed zebrafish larvae a high-fat diet (HFD) to drive liver fat accumulation (first hit). Next, we exacerbated liver-specific inflammation using a transgenic line (*fabp10-CETI-PIC3*) that induces the expression of proinflammatory cytokines following induction with doxycycline (second hit). These hits promoted fat accumulation and liver inflammation, as demonstrated by the high expression of inflammatory cytokines, macrophage infiltration, stress induction, and hepatic lipid droplet accumulation. Furthermore, zebrafish in this paradigm showed deranged glucose metabolism. To validate a small-molecule screening approach, we treated HFD-fed fish with pioglitazone, a drug shown to be beneficial for NAFLD in humans, and measured a sharp reduction in liver lipid accumulation. These results demonstrate new utility for zebrafish in modeling early NAFLD pathogenesis and demonstrate their feasibility for in vivo screening of new pharmacological interventions.

## 1. Introduction

In the United States, the number of NAFLD cases is projected to expand to 100.9 million in 2030, and the global prevalence is estimated at around 25% [1]. NAFLD comprises a broad spectrum of liver damage, which can range from macrovesicular steatosis to steatohepatitis (NASH), fibrosis and liver injury to cirrhosis, and hepatocellular carcinoma (HCC) [2]. Compared to the incidence of HCC in other liver diseases, a larger percentage of HCCs that arise in NASH occur before patients are cirrhotic, leading to larger and less treatable tumors [3]. Although there has been a growing interest in learning the pathogenesis of NAFLD and identifying key steps in development that could help stop the progression of the disease, no cure and few therapies have been developed.

There is a multiple-hit theory for the pathogenesis of NAFLD [4,5]. An initial “hit” comprises an insulin-resistant state that results from a predisposition to inflammation in the adipose tissue in the setting of a hypercaloric diet rich in fats and carbohydrates. The development of insulin resistance drives an increase in circulating free fatty acids, which may be sequestered in the liver via lipogenesis. This lipid accumulation, along with the presence of chronic inflammation, comprises a second “hit” in the pathogenesis of NAFLD. Recent studies in mice have revealed that hepatocyte inflammation may be an important link between the initial metabolic stress and subsequent hepatocyte death and stimulation of fibrosis in NASH [6,7]. Hepatic fibrosis is caused by the activation of hepatic stellate cells and myofibroblasts that leads to the subsequent deposition of a fibrous extracellular matrix [8]. Finally, through the expression of proinflammatory cytokines, such as IL-1β and IL-18, apoptosis is promoted.

To unravel the mechanisms of NAFLD pathogenesis, the majority of animal studies have utilized rodent models, such as mice and rats [9]. While these models have indeed provided a means to a deeper understanding of the roles of diet and overnutrition in the pathogenesis of NAFLD, they come with many limitations. These include cost, time and scale limitations, difficulty of in vivo pathological studies, and impracticality of *in vivo* small molecule screening. Recent studies have shown that lipid metabolism in zebrafish is similar to that in humans [10]. The benefits of using zebrafish for studying NAFLD include low cost, fast maturation, ease of genetic modification, and feasibility of experimental manipulations with respect to treatment paradigms [11].

Zebrafish serve as a physiologically relevant model system to study NAFLD owing to the similarity of the zebrafish hepato-pancreatic-biliary anatomy to humans [12]. Furthermore, at early developmental stages, the yolk strongly expresses apolipoproteins to metabolize the yolk [13]. Similar to mammals, microsomal triglycerides transfer protein play a critical role in lipid transport in zebrafish [14]. The zebrafish also express cholesteryl ester transfer protein (CETP) and are susceptible to atherosclerosis, making them attractive models for studying this disorder [15]. Apart from these physiological similarities, zebrafish also possess orthologues of critical lipid metabolic genes including microsomal TG transfer protein (*mttp*), fatty acid transport protein (*slc27a*), and acyl-CoA synthetase (*acsl*) gene families, as well as the LDL receptor (*ldlr*). In addition, the expression pattern of these genes appear to be comparable to humans [16].

In addition, the transparency and whole-body real-time monitoring make the larval zebrafish a promising model to study NAFLD [17]. Although there have been a few studies that have tried to utilize zebrafish for studying NAFLD pathogenesis using treatment paradigms similar to rodent models [11,18], there are no zebrafish models that demonstrate how inflammation plays a role in the pathogenesis of this disease. Recently, we established a model for tissue-specific, titratable cytokine induction in zebrafish [19], which may serve as a powerful animal model for studying diseases with a maladaptive inflammatory component. The Cre-Enabled Tetracycline Inducible transgenic system (CETI-PIC3) provides for liver-restricted overexpression of the cytokines IL1β, TNFα, and IFNγ, when crossed to the liver-specific *fabp10:Cre* zebrafish line. This system is novel and expedient because it models the inflammatory component on NAFLD and studies the downstream effects of cytokine induction of NAFLD.

## 2. Materials and Methods

### 2.1. Animal Experiments

Zebrafish (*Danio rerio*) were spawned and raised at 28.5 °C under standard laboratory conditions approved by the University of Chicago and the Indiana University Institutional Animal Care and Use Committees. The following established transgenic zebrafish lines were utilized: *Tg(mpeg:GFP)^gl22^*, *Tg(fabp10:dsred*, *ela3l:EGFP)^gz12^* (referred to as 2CLIP throughout for 2-Color Liver and Pancreas), and *Tg(CETI-PIC3)^iu15^*. A novel *Tg(fabp10:cre)* line was generated using transgene constructs described previously [20]. *Tg(fabp10:cre)* and *Tg(CETI-PIC3)^iu15^* fish were used together to generate “Li-PIC3” zebrafish for liver-specific induction of zebrafish orthologues of the cytokines IL1β, TNFα, and IFNγ. *Tg(mpeg:GFP)* fish were utilized for macrophage visualization and *Tg(fabp10:dsRed)* fish were used for liver visualization. All embryos were collected at spawning and incubated at 28.5 °C in egg water-filled (0.1% instant ocean salt, 0.0075% calcium sulfate) petri dishes. After gastrulation stages, 0.003% 1-Phenyl-2-thiourea (PTU; Acros #207250250, New Jersey, USA) supplementation in egg water was used to prevent pigmentation in all embryos and larvae.

### 2.2. Inflammatory Liver Model

*Tg(CETI-PIC3)^iu15^* fish were crossed with *Tg(fabp10:cre)* fish to generate CETI-PIC3/fabp10:cre embryos that overexpress cytokines IL-1β, TNFα, and IFNγ following treatment with doxycycline. Ten CETI-PIC3/fabp10:cre embryos and ten *Tg(fabp10:cre)* control larvae at 7 days post fertilization (dpf) were incubated in 3 mL of 2.5 µg/mL doxycycline in egg water for 24 h. For time-point studies, 7 dpf larvae were treated for 0 h, 3 h, 6 h, 12 h, or 24 h. Larvae were fixed at 8 dpf with 3% formaldehyde in PEM buffer (0.21 M PIPES, 1 mM MgSO_4_, 2 mM EGTA, and pH 7) at 4 °C overnight.

### 2.3. High-fat Diet Feeding and Lipid Staining

A 5% chicken egg solution in egg water (High-fat diet, HFD) was prepared by mixing 1 mL chicken egg in 19 mL egg water. BODIPY analog (Invitrogen # D3922, Carlsbad, CA, USA) was added to 10 mL of the 5% chicken egg solution and vortexed for 30 s for a final concentration of 6.4 µM of BODIPY-FL. Twenty 5 dpf larvae were incubated in 4 mL of the chicken egg solution for 2 h in the dark at 28.5 °C and washed in egg water following feeding. Feeding was repeated at 7 dpf, and larvae were fixed at 8 dpf with 3% formaldehyde in a PEM buffer at 4 °C overnight.

### 2.4. Pioglitazone Treatment

Pioglitazone (PIO; Thermo Fisher Scientific # 41-245-0, Waltham, MA, USA) was added to egg water at 5 or 10 µM along with the HFD. After 2 h of HFD treatment, the larvae were washed with egg water and incubated again with the same concentration of PIO until the following HFD treatment, and then to the end of the experiment. PIO-treated embryos and controls were stained with BODIPY dissolved at 40 µM in 0.1% DMSO for 1 h, then washed extensively for 1 h and fixed in 3% formaldehyde in buffered saline at 4 °C overnight. Larvae were micro-dissected to remove skin and any residual yolk, then mounted in Vectashield (Vector Labs, Burlingame, CA, USA) and imaged with confocal microscopy. One microliter optical sections were collected and analyzed by NIH FIJI software using thresholding, contiguous area measurement, and analyze particles functions. The index for comparing fatty liver conditions was calculated by dividing the number of lipid droplets by the pixel area marked by *fabp10:dsRed* (2CLIP) fluorescence in each sample, and is displayed in arbitrary units.

### 2.5. Free Glucose Assay

Glucose colorimetric assays were performed to measure free glucose levels in larvae. Glucose colorimetric assays (BioVision #K686, Milpitas, CA, USA) were performed by homogenizing 20 larvae within 500 µL of assay buffer and then following the manufacturer’s protocol. Results were measured using a SpectraMax M5 multiwell plate reader (Molecular Devices, Sunnyvale, CA, USA).

### 2.6. Combination Inflammatory Liver and High-Fat Diet Feeding Model

Li-PIC3 larvae were fed HFD at 5 dpf and 7 dpf as described above. Following HFD feeding at 7 dpf, larvae were incubated in 3 mL of 2.5 µg/mL Doxycycline in egg water for 24 h. Larvae were then fixed with 3% formaldehyde in a buffered saline at 4 °C overnight.

### 2.7. Detection of Reactive Oxidative Species

To detect Reactive Oxidative Species (ROS), zebrafish were incubated in 10 µM CellRox Deep Red (Invitrogen #C10422, Carlsbad, CA, USA) in egg water in the dark at 28.5 °C for 1 h. Larvae were washed with PBS and fixed immediately after with 3% formaldehyde in buffered saline at 4 °C overnight, as described previously [21].

### 2.8. Immunofluorescence Staining

Antibody staining was performed as described [22]. Briefly, whole mount zebrafish samples were stained with primary antibodies: mouse anti-TNFα (1:50; Abcam #52B83, Cambridge, UK), guinea pig anti-insulin (1:200, Invitrogen #180067, Carlsbad, CA, USA), rabbit anti-cleaved caspase 3 (1:100; Cell Signaling Technologies #9661S, Danvers, MA, USA), mouse anti-Prox1 (1:50, Developmental Studies Hybridoma Bank, AB_2619013, Iowa City, IA, USA), and chicken anti-GFP (1:200, Aves labs #GFP-1010, Davis, CA, USA). Primary antibodies were detected with complementary Alexa-conjugated secondary antibodies (1:500, Jackson ImmunoResearch, West Grove, PA, USA). DNA was stained with TO-PRO3 (1:500, Thermo Fisher #T3605, Waltham, MA, USA) or DAPI (1:400, Thermo Fisher #62248, Waltham, MA, USA). After staining, larvae were mounted on slides in VECTASHIELD (Vector Labs H-1000). Confocal imaging was performed with a Zeiss LSM700 or Nikon A1 microscope. Images of livers were analyzed using NIH Fiji software. Images following immunofluorescence staining were used to verify the presence of TNFα and H2BGFP in livers and to count infiltrating macrophages.

### 2.9. Quantitative Real-Time PCR

RNA isolation and reverse transcription were performed using miRNeasy and miScript II RT kits according to the manufacturer’s protocol (Qiagen, Hilden, Germany). Quantitative real-time PCR (qPCR) was performed using the miScript SYBR Green PCR Kit (Qiagen, Hilden, Germany) and a Mastercycler realplex instrument (Eppendorf, Hauppage, NY, USA). Using the comparative Ct method as described [23], mRNA levels of IL-1β, TNFα, and IFNγ were determined relative to mRNA levels of β-actin.

### 2.10. Statistical Analysis

Statistical analyses were performed using GraphPad Prism Version 9.3 (GraphPad Software, La Jolla, CA, USA). Student’s *t*-tests were used for comparison between experimental and control groups. One-way ANOVA with Tukey’s post-test for multiple comparisons was used when comparing more than two groups. A *p*-value of ≤0.05 was considered significant (indicated with *) for all analyses. Lesser *p*-values of ≤0.01, ≤0.001, and ≤0.0001 were indicated with **, ***, and ****, respectively.

## 3. Results

### 3.1. Models Exhibit Cytokine Expression and Lipid Accumulation in the Hepatocytes

**Figure 1:**
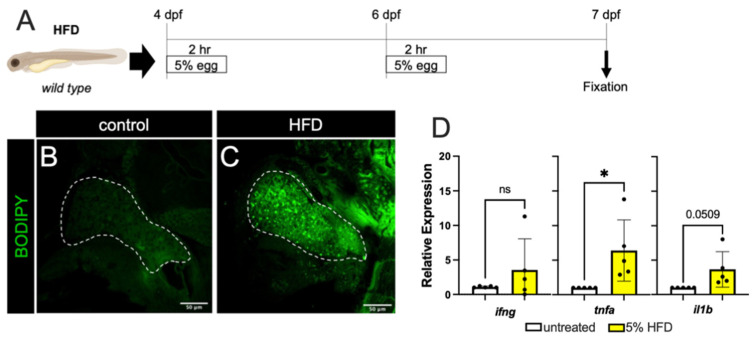
High fat diet treatment. (**A**) The schematic for high-fat diet feedings of the zebrafish larvae. (**B,C**) BODIPY labeling of unfed (**B**) and 5% High-fat Diet (HFD) fed larvae (**C**). There is minimal accumulation of lipid droplets in the liver of unfed larvae (outlined by dashed white line), while HFD treatment resulted in significant accumulation of lipid droplets in hepatocytes. (**D**) The larval mRNA level of *tnfa* only is significantly increased in response to HFD. Expression analyses were performed on whole larvae. Statistical analyses by students *t*-test, *n =* 4–5 clutches with at least 15 embryos per clutch. ns—not significant,* *p* < 0.05.

First, to produce a state of overnutrition in our model, we used a high-fat feeding paradigm (depicted in Figure 1A) wherein zebrafish larvae were immersed in a 5% homogenized chicken egg solution for 2 h, at both 4 days post-fertilization (dpf) and 6 dpf. This high-fat diet (HFD) treatment of larvae resulted in fat accumulation in the liver, as revealed by BODIPY labeling of lipid droplets in hepatocytes (Figure 1B,C). To determine if an inflammatory state was promoted, we measured the expression of the zebrafish orthologues of the pro-inflammatory cytokines IFNγ (*ifng*), TNFα (*tnfa*), and IL-1β (*il1b*) using quantitative PCR (qPCR) of whole embryo cDNA preparations and found a mild but statistically significant increase in *tnfa* only (Figure 1D). These results indicate that dietary lipids are being consumed by the larvae, that these are processed and trafficked to the hepatocytes, and this may induce a mildly inflamed state.

**Figure 2:**
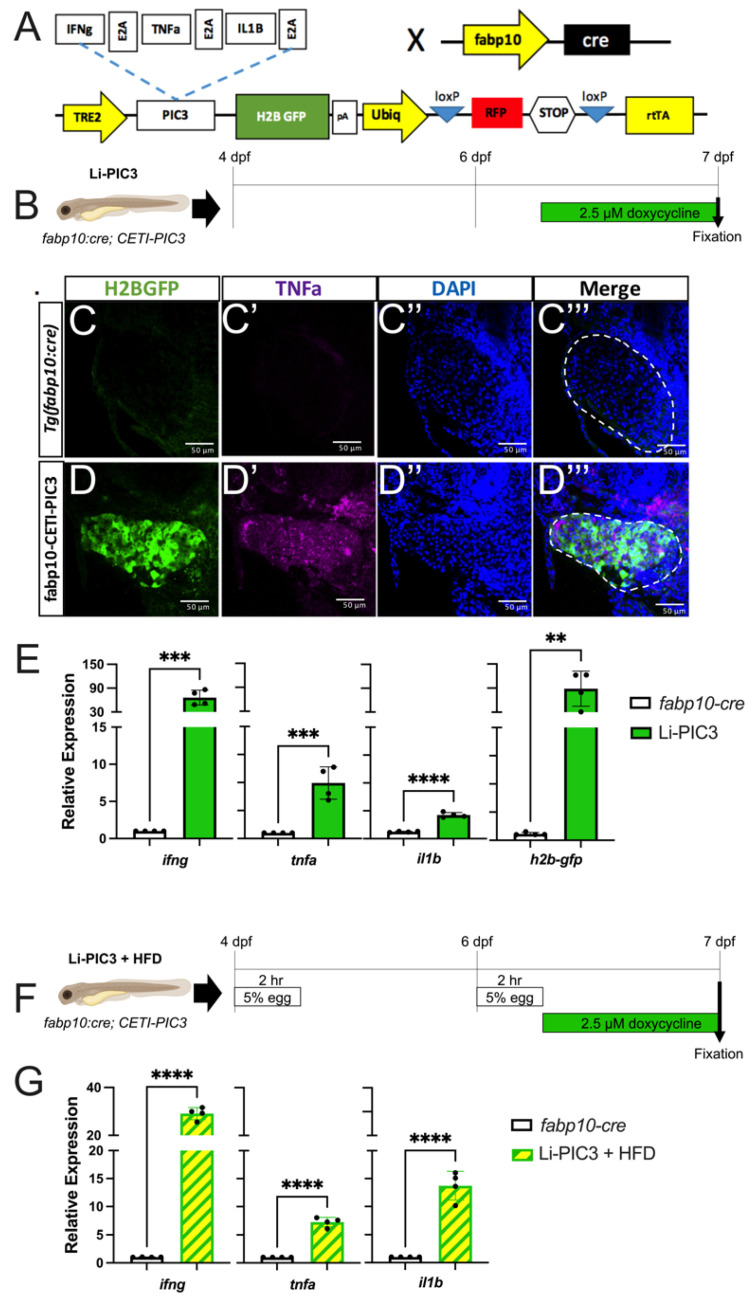
Hepatic induction of proinflammatory cytokines. (**A**) Diagram of the CETI-PIC3 transgene that is enabled in a hepatocyte-specific manner by *fabp10:cre.* (**B**) Experimental scheme for induction of the PIC3 cytokines at 6 dpf. (**C**–**D’’’**) Immunostaining of 7 dpf *Tg(fabp10:cre)* control larvae (**C**) and Li-PIC3 larvae (**D**) for GFP (green, C,D), TNFα (magenta, **C’**,**D’**), DNA (blue, **C’’**,**D’’**), and the merged images (**C’’’**,**D’’’**). The PIC3 cassette was strongly induced by doxycycline, as evidenced by both GFP and TNFα labeling in the liver (outlined by dashed white line). (**E**) The levels of *ifng*, *tnfa*, *il1b*, and *H2B-GFP* are all increased after doxycycline induction in the Li-PIC3 embryos as compared to *Tg(fabp10:cre)* control embryos. (**F**) Diagram for combined HFD followed by dox induction in zebrafish larvae. (**G**) The levels of *ifng*, *tnfa*, and *il1b* are all increased in Li-PIC3/HFD larvae as compared to *Tg(fabp10:cre)* control embryos. Expression analyses were performed on whole larvae. Statistical analyses by students *t*-test, *n =* 4–5 clutches with at least 15 embryos per clutch. ** *p* < 0.01, *** *p* < 0.001, **** *p* < 0.0001.

Next, we sought to elicit a hyper-inflammatory response in the liver by directly inducing the misexpression of IFNγ, TNFα, and IL1β using the Cre-Enabled Tetracycline Inducible transgenic zebrafish line (CETI-PIC3) that we previously developed [19]. For this approach, CETI-PIC3 transgenic zebrafish were bred with *fabp10:cre* transgenic animals to generate *fabp10:Cre*^+^; CETI-PIC3^+^ (henceforth Li-PIC3), which express cre only in hepatocytes [24] (Figure 2A). Thus, in these doubly transgenic animals, the hepatocyte-restricted transcription of cytokines and a translationally-linked fluorescent H2B-GFP marker located downstream from the cytokines, is enabled after recombination. Treatment with 2.5 µg/mL doxycycline from 6 dpf to 7 dpf (schematized in Figure 2B) induced the expression of the three cytokines and the H2B-GFP marker in the liver (Figure 2C–E). Hepatocyte expression of H2B-GFP and TNFα was confirmed via immunofluorescent staining (Figure 2C,D). Furthermore, expression of the cytokines and H2B-GFP was examined by qPCR in whole embryo cDNA preparations, and each showed significantly elevated expression relative to *fabp10:cre* controls.

Lastly, we combined these Li-PIC3 and HFD-feeding approaches to generate a multiple-hit model of NAFLD (Li-PIC3+HFD, schematized in Figure 2F). Larvae were immersed in 5% chicken egg solution for 2 h at 4 and 6 dpf, then treated with 2.5 µg/mL doxycycline from 6–7 dpf. Again, we used qPCR to measure transcriptional changes in cytokine expression and found that the levels of *ifng, tnfa,* and *il1b* mRNA were all significantly increased as compared to *Tg(fabp10:cre)* control larvae that were not fed HFD (Figure 2G).

### 3.2. NAFLD Induction Promotes Infiltration of Macrophages

**Figure 3:**
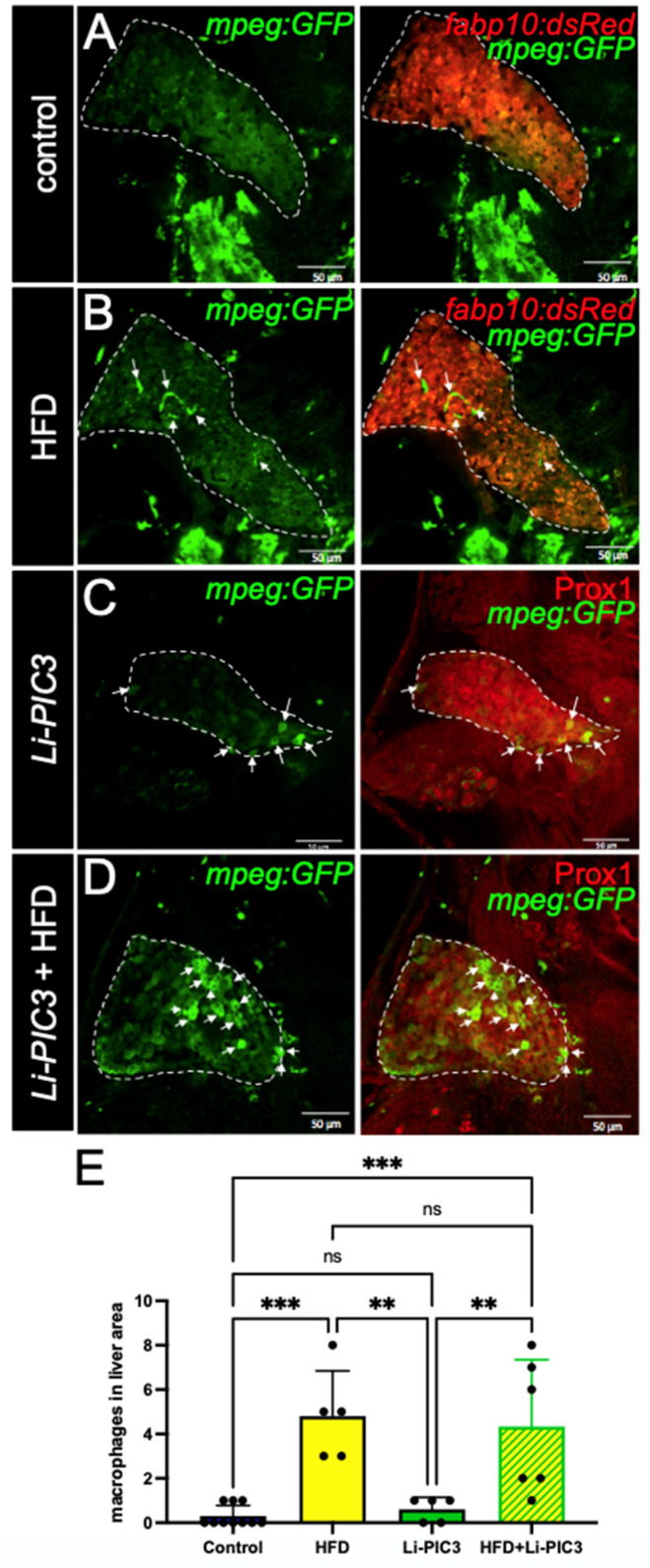
Macrophage infiltration is increased in HFD, Li-PIC3, and Li-PIC3+HFD larvae. (**A**–**D**) 7 dpf larvae labeled with GFP immunostaining to reveal macrophages and either a 2CLIP transgene (**A,B**) or immunolabeled with Prox1 antibody (**C,D**) to reveal hepatocytes. Infiltrating macrophages are indicated by white arrows. (**E**) Quantification of macrophages within the liver boundary reveals a statistically significant increase with HFD and Li-PIC3+HFD treatments. Statistical analysis by one-way ANOVA, ns-non significant, ** *p* < 0.01, *** *p* < 0.001.

NAFLD progression is characterized by the infiltration of macrophages [25]. To study the phenotype of macrophage infiltration in our NAFLD model, we bred the *Tg(mpeg:GFP)* allele into our Li-PIC3 transgenic fish to mark macrophages with green fluorescence. Whereas macrophages were rarely observed within the liver boundaries at 7 dpf under control conditions (Figure 3A), they were often observed in the livers of HFD, Li-PIC3, and Li-PIC3+HFD larvae (Figure 3B–D). When infiltrating macrophages were quantified, we calculated a statistically significant elevation in hepatic macrophage infiltration for HFD and Li-PIC3+HFD larvae relative to controls (Figure 3E).

### 3.3. NAFLD Induction Leads to ROS Accumulation and Liver Damage

**Figure 4:**
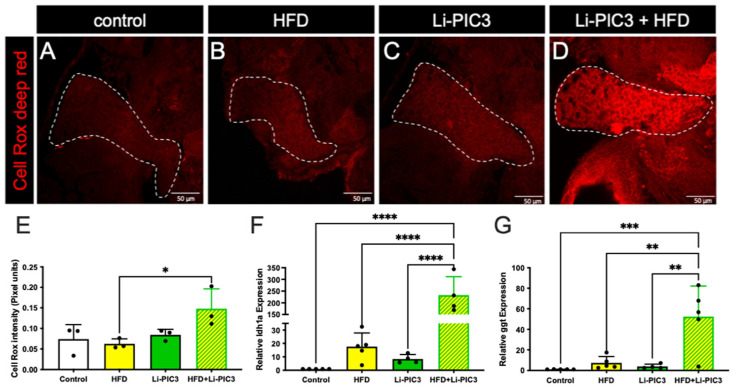
ROS mediated stress is induced in livers of zebrafish fed a high-fat diet and liver-specific cytokine induced embryos. (**A**–**D**) Cell Rox Deep Red staining used to visualize ROS-mediated stress in the livers of control, HFD-fed, Li-PIC3, and Li-PIC+HFD larvae. (**E**) Quantification of Cell Rox intensity demonstrated an increase in only Li-PIC3+HFD embryos as compared to *Tg(fabp10:cre)* controls, while the comparison to controls trended towards significance (*p* = 0.776). (**F**) Quantification of *ldh1a* mRNA levels by qPCR (**G**) Quantification of *ggt* mRNA levels by qPCR. Both ldh1a and ggt are elevated in the combined Li-PIC3+HFD condition. Statistical analysis was by one-way ANOVA, *n* = 4–5 per condition; * *p* < 0.05, ** *p* < 0.01, *** *p* < 0.001, **** *p* < 0.0001.

To assess reactive oxygen species (ROS)-mediated stress in the liver, which is a common feature of NAFLD, cohorts of HFD-fed, Li-PIC3, and Li-PIC3+HFD larvae, and controls were each stained with CellRox Deep Red, an indicator of ROS (Figure 4A–D). When the intensity of CellRox staining was quantified, we detected no increase with either of the single treatments, HFD or Li-PIC3 (Figure 4E). However, we detected nearly a 2-fold increase in ROS under the combined Li-PIC3+HFD experimental conditions. Next, in humans with liver damage, gamma-glutamyl transferase (GGT) and lactate dehydrogenase (LDH) proteins are released into circulation [26,27], and their levels may be used as a diagnostic benchmark for determining the extent of the injury. Accordingly, we reasoned that *ggt* and *ldh1a* mRNA expression levels would be correspondingly elevated in the hepatocytes of damaged livers, mirroring their corresponding elevated serum protein levels; this would permit measurement of *ggt* and *ldh1a* in zebrafish larvae as a surrogate approach to quantifying the extent of hepatocyte injury where it is not possible to assay serum. Thus, we measured the mRNA expression levels of *ldh1a* and *ggt* using qPCR. (Figure 4F,G). Whereas larvae subjected to each of the single experimental stresses showed no significant evidence of liver injury, those in the combined Li-PIC3+HFD condition showed striking elevations of *ggt* (>50-fold) and *ldh1a* (>230-fold).

### 3.4. Association of NAFLD with Insulin Resistance

**Figure 5:**
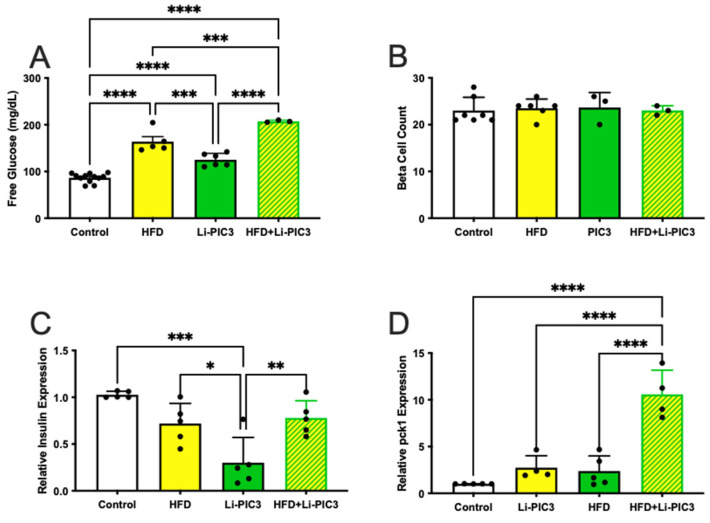
Hyperglycemia is induced without loss of β cells in HFD, Li-PIC3, and Li-PIC3+HFD larvae. (**A**) Blood glucose levels for *Tg(fabp10:cre),* HFD, Li-PIC3, and Li-PIC3+HFD larvae. (**B**) β cell count in *Tg(fabp10:cre),* HFD, Li-PIC3, and Li-PIC3+HFD larvae. (**C**) Transcript levels for insulin *Tg(fabp10:cre)*, HFD, Li-PIC3, and Li-PIC3+HFD larvae. (**D**) Levels of *pck1*, a marker of gluconeogenesis, in *Tg(fabp10:cre),* HFD, Li-PIC3, and Li-PIC3+HFD larvae. Statistical analysis was by one-way ANOVA, n = 3–9; * *p* < 0.05, ** *p* < 0.01, *** *p* < 0.001, **** *p* < 0.0001.

In humans, NAFLD is strongly associated with insulin resistance, obesity, and type 2 diabetes [28]. We hypothesize that treatment with HFD, Li-PIC3, and their combination is associated with increasing levels of pancreatic β cell dysfunction/insufficiency as reflected by increasing systemic glucose levels. To measure glucose levels in zebrafish larvae, cohorts of like-treated larvae were pooled and homogenized, and free glucose levels were measured via colorimetric assay as previously described [22]. We found that glucose was elevated relative to controls under all conditions tested (Figure 5A), though the highest levels were measured in larvae subjected to the Li-PIC3+HFD combined treatment. To determine if this relative deficit in β cell function was related to a change in the absolute quantity of β cells, we counted β cells using confocal microscopy. No differences were observed among any of the treatments (Figure 5B). Next, we measured the expression of mRNA to determine if insulin levels produced by the β cells were responsible for the change in glucose levels. We found small but significant variation in insulin expression, with the lowest levels in the single Li-PIC3 larvae (Figure 5C). Lastly, we measured the expression of phosphoenolpyruvate carboxykinase (*pck1*) in zebrafish as a measure of insulin signaling; pck1 is expressed in the liver, where it catalyzes the first committed step in gluconeogenesis, and its expression is suppressed by insulin. We found that pck1 levels remained unchanged with the single perturbations of HFD and Li-PIC3 but were elevated nearly 10-fold with the combination Li-PIC3+HFD treatment (Figure 5D).

### 3.5. Pioglitazone Treatment Abates Steatosis

**Figure 6:**
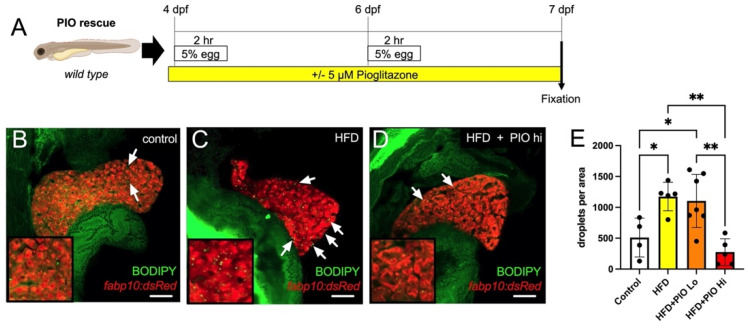
Pioglitazone treatment reduces lipid droplet accumulation in the liver. (**A**) Experimental scheme of PIO treatment. Larvae were immersed in PIO+ 0.1% DMSO for 1 h prior to the first HFD treatment and remained in PIO throughout. (**B**–**D**) Representative optical slices of BODIPY-stained 2CLIP zebrafish liver depicting (**B**) untreated controls, (**C**) HFD-fed larvae, and (**D**) HFD-fed + PIO hi (5 µM) treated larvae; hepatocytes are labeled in red via expression of the transgene *fabp10:dsRed* and examples of green-labeled lipid droplets are indicated by arrows. (**E**) Quantification of lipid droplets in hepatocytes area as executed with FIJI software. Statistical analysis was by one-way ANOVA; * *p* < 0.05, ** *p* < 0.01. Scale bar = 50 µm.

The thiazolidinedione PPARγ agonist Pioglitazone (PIO) stimulates hepatic fatty acid oxidation and improves insulin sensitivity and reduces steatosis in NAFLD patients [29,30]. Thus, to test the utility of our zebrafish model in screening for pharmacological interventions, we treated HFD-fed zebrafish larvae in combination with PIO between 4 and 7 dpf (Figure 6A). BODIPY-labeled neutral lipid droplets were quantified per pixel area of liver tissue in confocal optical sections using FIJI software. As expected, HFD treatment significantly increased fat deposits in the liver (511.0 ± 315.2 in control unfed larvae versus 1174 ± 230.7 in HFD-fed, *p* = 0.03) (Figure 6B,C,E). While a lower dose of 1µM PIO showed no significant effect on lipid accumulation versus HFD alone (1104 ± 430.7 *p* = 0.982), treatment with 5 µM PIO significantly reduced the quantity of lipid droplets (275.5 ± 215.5, *p* = 0.0022), returning them to control levels (Figure 6 D,E).

## 4. Discussion

Lipid accumulation and inflammation in the liver both play a critical role in the pathogenesis of NAFLD [31]. Most in vivo studies of NAFLD pathogenesis using animal models have been performed in rodent models and have been largely dependent on diet and obesity [11]. The methionine and choline-deficient (MCD) diet is high in sucrose and fat but lacks methionine and choline, which are essential for hepatic β-oxidation; rats and mice fed this diet develop features of NAFLD, including steatosis and fibrosis [32]. However, one major disadvantage to the MCD model is that insulin, leptin, and glucose levels lead to greater insulin sensitivity, the opposite of that seen in NAFLD patients [33]. Another study demonstrated that mice fed a high-fat diet to the point of obesity developed insulin resistance and hepatic damage. However, these mice did not have as severe liver damage as those in the MCD model [34]. Thus, although many models can lead to lipid accumulation in the liver, not all replicate aspects of the pathology of human NAFLD.

In this study, we utilized the strengths of the zebrafish model to address these limitations. Due to substantial genetic, anatomical, and physiological similarities between the hepato-pancreatic systems of humans and zebrafish, these aquatic organisms are quite suitable for studying a metabolic disorder such as NAFLD. Previous studies by others have used zebrafish embryos, larvae, and adults to model NAFLD through diets with high cholesterol, high fructose, or by simply excessively feeding for 10 days. Interestingly, the development of steatosis has been found to be most severe in the excessive feeding models [11]. Other zebrafish studies have shown that feeding zebrafish larvae a high-fat diet and a high-fat plus high-cholesterol diet induces simple steatosis, with the high-fat plus high-cholesterol diet causing more severe steatosis [18]. However, these models do not completely mimic human NAFLD pathogenesis in which hyper-inflammation plays a critical role as well [35].

In our study, we subjected zebrafish larvae to a “two-hit” paradigm, wherein they were first fed a lipid-rich diet to excess, followed by induction of cytokines using our Liver-CETI-PIC3 system. We studied macrophage infiltration using the *Tg(mpeg:GFP)* line that we have well-characterized for studying migration and infiltration of these immune cells in other inflammation models [36,37]. Curiously, while the animals subjected to the HFD alone exhibited a clear inflammatory response, those only mis-expressing cytokines hepatically via Li-PIC3 did not. Importantly, the addition of Li-PIC3 cytokine expression to the HFD treatment led to an enhanced character of the inflammation in the liver, modeling a more severe state of steatohepatitis. For instance, increased ROS, decreased activity of detoxifying enzymes, and an increase in net oxidative stress are strongly associated with NAFLD (reviewed in [38,39]). The combination of HFD with Li-PIC3 resulted in increased ROS in the liver as compared to HFD alone, while other conditions were indistinguishable from one another. Additionally, expression of *ldh1a* and *ggt*, whose expression has been shown to be elevated in some liver diseases [40,41] and used here as surrogate measures of liver cell dysfunction, were unchanged from controls with either HFD or Li-PIC3 treatments alone. However, expression of these markers was dramatically upregulated (200- and 50-fold for *ldh1a* and *ggt*, respectively) in the Li-PIC3+HFD animals. Lastly, we observed elevated systemic free glucose levels in larvae under all experimental conditions, and whose levels were highest in Li-PIC3+HFD larvae despite normal numbers of pancreatic beta cells and normal insulin expression. This, together with the sharply increased expression of phosphoenolpyruvate carboxykinase (*pck1*) only under Li-PIC3+HFD conditions, suggests a deregulation of glucose metabolism mirroring aspects of human metabolic syndrome. As insulin potently suppresses the transcription of *pck1* within minutes, and as pck1 is a key rate-limiting enzyme for gluconeogenesis, this finding is consistent with the interpretation that Li-PIC3+HFD larvae are highly insulin resistant. Taken together, our data suggest that excess dietary lipids are trafficked to the liver and elicit a generally benign immune infiltration of macrophages. Upon hepatic expression of proinflammatory cytokines by Li-PIC3, a local milieu is generated, which drives oxidative stress and triggers insulin resistance and likely other cellular dysfunctions. It will be important in future studies to characterize the infiltrating macrophages and to define their activation states, i.e., are they classically activated, and how are the macrophages of any state of activation integral to the observed NAFLD phenotypes in this model.

As presented in this study, our model system is unique and powerful for studying NAFLD in that it combines fat accumulation with targeted inflammation in the liver. In the future, our model can be modified to examine different degrees of steatosis. Furthermore, because the CETI-PIC3 system is titratable, different levels of severity of NAFLD such as Non-Alcoholic Steatohepatitis can be modeled through increased and or chronic cytokine induction. In addition, we fed zebrafish larvae a high-fat diet consisting of 5% chicken egg in PTU water, but alternative diets may be fed to zebrafish to model NAFLD-like outcomes. These might include combination high-fat and high-cholesterol diets, or high-fat/low-protein diets. Protein deficiency is associated with NAFLD and NASH because proteins are important for hepatocyte regeneration and the prevention of fat deposits in the liver [42]. Finally, in this study, while a HFD is used as the first “hit” and cytokine-mediated inflammation the second parallel “hit” for pathogenesis of NAFLD, a model in which cytokines are first induced and a HFD is fed afterward, can also be studied. It is important to note that NAFLD pathogenesis is a complex metabolic disorder that involves other factors including hormonal regulation [43], gut microbiota dysbiosis, and bile acid abnormalities [44] that are not easily recapitulated at the larval stages of zebrafish. For that, supplementary studies in more complex physiological conditions including adult staged zebrafish or mammalian models are necessary.

Lastly, we have also demonstrated that zebrafish can be utilized as an efficient and facile platform for investigating pharmacological agents that may be translatable to treat NAFLD patients. Here, we used pioglitazone as a proof of this concept, as it has been shown to improve NAFLD measures in human studies [45,46]. Pioglitazone binds to the PPARγ, a member of the nuclear receptor superfamily. PPARγ is known to play a role in glucose regulation and lipid metabolism [47]. Pioglitazone is metabolized by hydroxylation and oxidation in the liver to form different metabolites [48]. Further studies could reveal whether NAFLD outcomes might be modulated by titrating the dosages of pioglitazone.

## 5. Conclusions

In conclusion, we have demonstrated that our zebrafish model can be utilized for studying the early pathogenesis of NAFLD, especially for testing the 2-hit theory of NAFLD pathogenesis. Our studies show the efficiency and feasibility of zebrafish as a platform in vivo for unbiased testing of novel small molecules that could impact the pathogenesis of NAFLD.

## Data Availability

All of the primary data in this manuscript are available on Mendeley Data (Mirmira, Raghavendra (2022), “A Novel 2-Hit Zebrafish Model to Study Early Pathogenesis of Non-Alcoholic Fatty Liver Disease”, Mendeley Data, V1, doi:10.17632/tw458tcx88.1).
